# Supplementary material for: SARS-CoV-2 RBD trimer protein adjuvanted with Alum-3M-052 protects from SARS-CoV-2 infection and immune pathology in the lung
Source: Nat Commun. 2021 Jun 11;12:3587. doi: 10.1038/s41467-021-23942-y (PMC8196016; doi:10.1038/s41467-021-23942-y)
Supplement: Supplementary file 1 — Supplementary Information [file 41467_2021_23942_MOESM1_ESM.pdf]

SARS-CoV-2 RBD Trimer Protein Adjuvanted with Alum-3M-052 Protects from SARS-CoV-2 infection and Immune Pathology in the Lung

Nanda Kishore Routhu[1,2,*], Narayanaiah Cheedarla[1,2,*], Venkata Satish Bollimpelli[1,2,*], Sailaja Gangadhara[1,2,*], Venkata Viswanadh Edara[1,3], Lilin Lai[1,3], Anusmita Sahoo[1,2], Ayalnesh Shiferaw[1,2], Tiffany M Styles[1,2], Katharine Floyd[1,3], Stephanie Fischinger[4], Caroline Atyeo[4], Sally A. Shin[4], Sanjeev Gumber[5], Shannon Kirejczyk[5], Kenneth H. Dinnon III[6], Pei-Yong Shi[7], Vineet D. Menachery[8], Mark Tomai[9], Christopher Fox[10], Galit Alter[4], Thomas H Vanderford[1], Lisa Gralinski[11], Mehul S. Suthar[1,2,3] and Rama Rao Amara[1,2,†]

[1]Emory Vaccine Center, Division of Microbiology and Immunology, Yerkes National Primate Research Center, Emory University, Atlanta, Georgia 30329, USA.

[2]Department of Microbiology and Immunology, Emory School of Medicine, Emory University, Atlanta, Georgia 30322, USA.

[3]Department of Pediatrics, Division of Infectious Diseases, Emory University School of Medicine, Atlanta, GA 30322, USA.

[4]Ragon Institute of MGH, MIT and Harvard, Cambridge, Massachusetts, USA.

[5]Division of Pathology, Yerkes National Primate Research Center, Emory University, Atlanta, Georgia 30329, USA.

[6]Department of Microbiology and Immunology, University of North Carolina, Chapel Hill, NC 27599, USA.

[7]Department of Biochemistry and Molecular Biology, The University of Texas Medical Branch, Galveston, TX, USA.

[8]Department of Microbiology and Immunology, The University of Texas Medical Branch, Galveston, TX, USA.

[9]3M Corporate Research Materials Laboratory, Saint Paul, MN, USA

[10]Infectious Disease Research Institute, Seattle, WA, USA

[11]Department of Epidemiology, University of North Carolina, Chapel Hill, NC 27599, USA.

Footnotes

[*]Contributed equally to this work

[†]**Correspondence:** Correspondence should be addressed to Dr. Rama Amara. Phone: (404) 727-8765; FAX: (404) 727-7768; E-mail: ramara@emory.edu

Supplementary Fig. 1

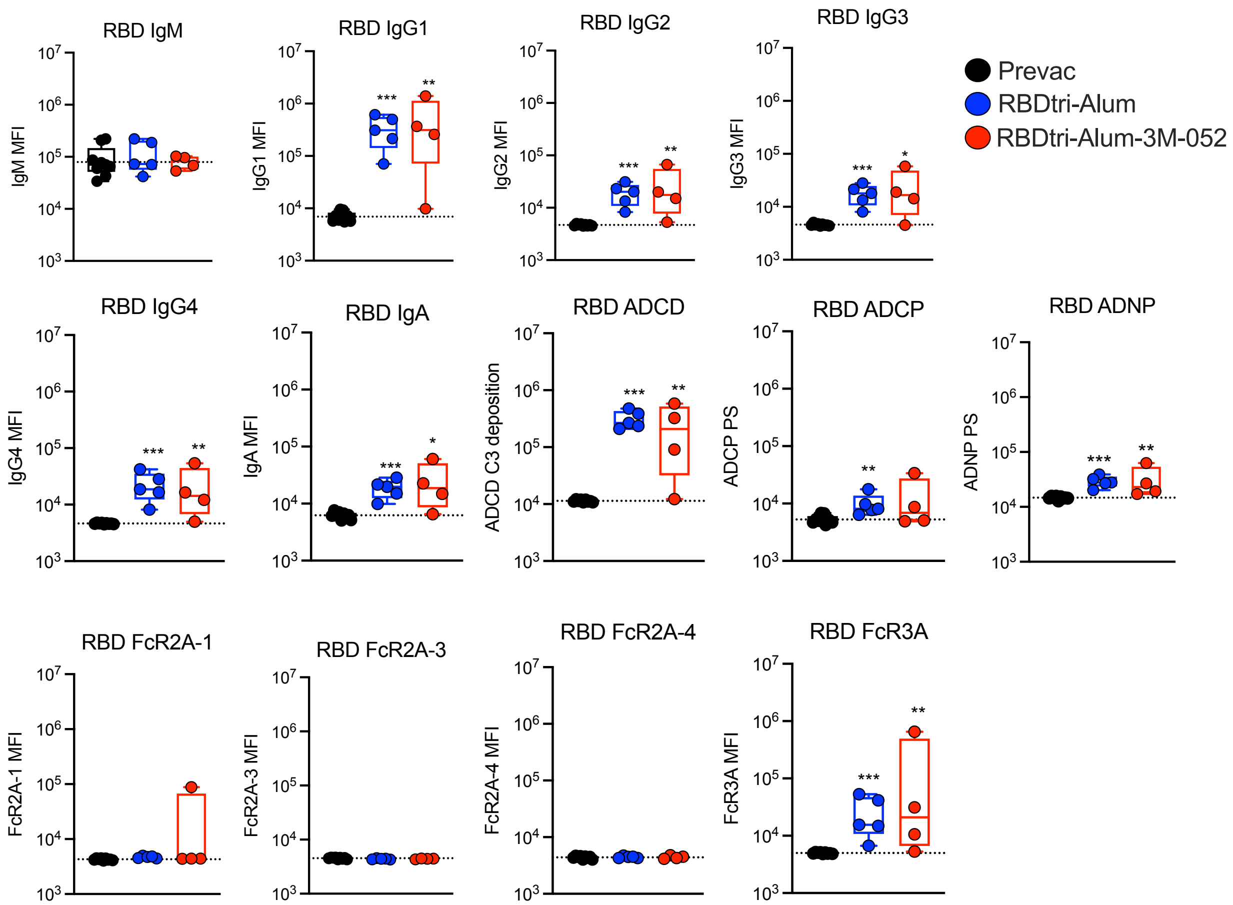

Supplementary Fig. 1: Antibody subclass, non-neutralizing antibody effector functions and FcγR binding activities of antibody induced after RBD trimer vaccination in macaques. Box plots showing SARS-CoV-2 RBD-specific systems serology measurements (antibody isotypes, antibody-dependent effector functions and FcγR binding) pre vaccination (prevac) and week 6 post-vaccination in alum (blue) or alum-3M-052 (red)) groups. Data for pre vaccination time point from both groups is shown together (black). Each filled circle represents an individual animal. Error bars show minimum to maximum. *p*-values indicate difference between prevac and post vaccination time points. Statistical differences were calculated using unpaired Mann-Whitney (two-tailed) test. ∗, $p<0.05$; **, $p<0.01$ and ***, $p<0.001$.

Supplementary Fig. 2

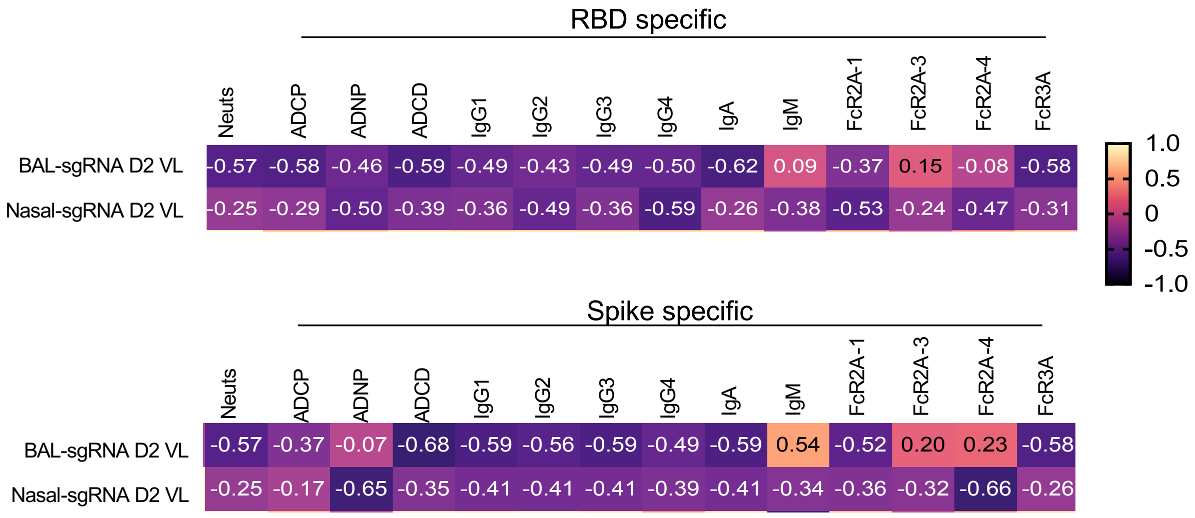

Supplementary Fig. 2: Correlations between Day 2 viral loads (BAL and Nasal) and antibody specificity and function. The correlation matrix was generated between Day 2 viral loads (BAL and Nasal) and respective antibody specificity or function. The color refers to r value scale shown on the right. The number in each cell indicate the actual r value. The Spearman rank test was used to perform correlation analysis. None of the correlations were statistically significant.

Supplementary Fig. 3

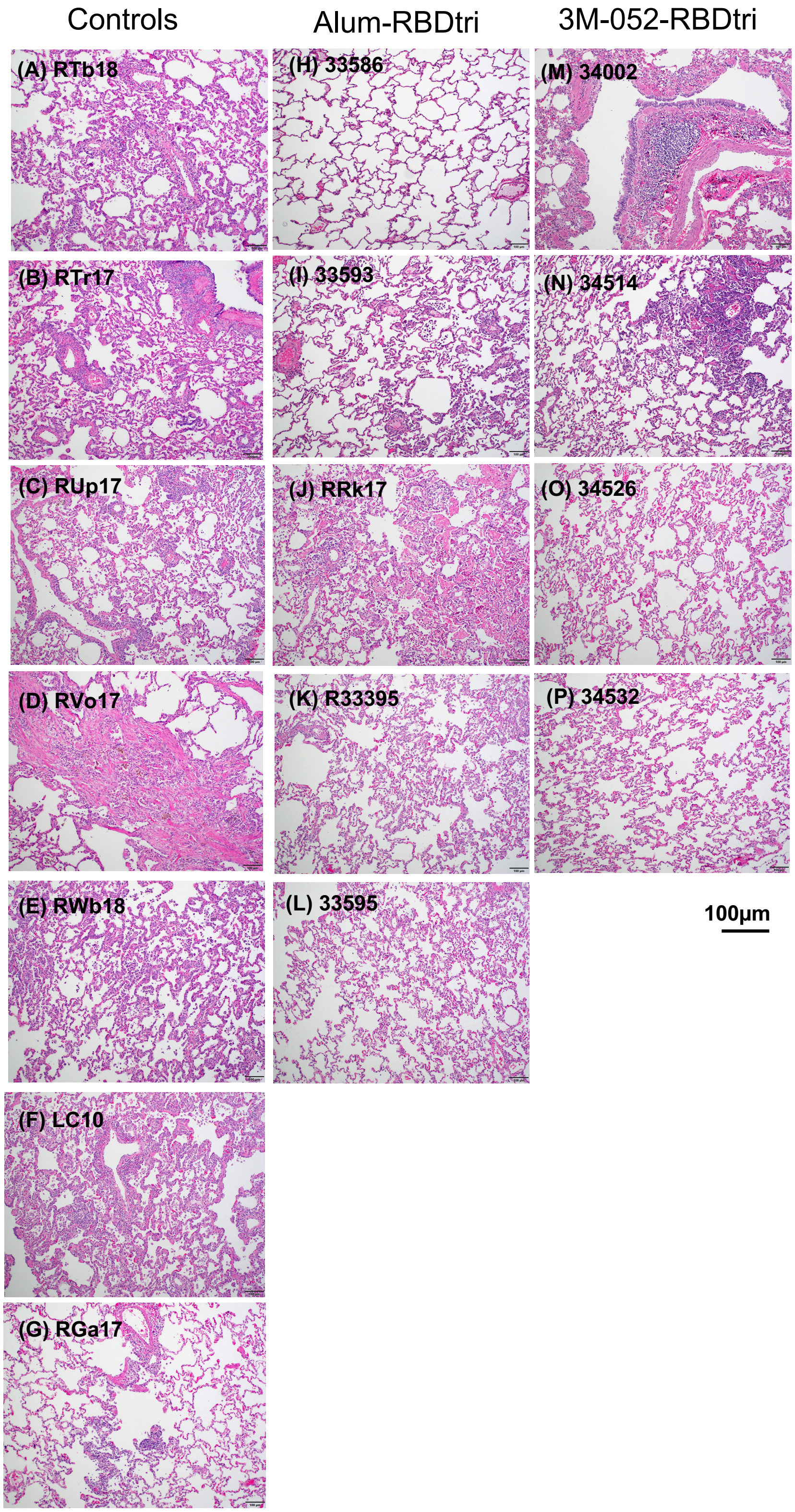

(A - G) Controls;
(H – L) Alum-RBDtri;
(M– P) 3M-052-RBDtri.

Supplementary Fig. 3: Post-challenge lung pathology in rhesus macaque. Lung Pathology of rhesus macaques infected with SARS-CoV-2 (Panels A-G) and vaccinated with Alum-RBDtri (Panels H-L) or 3M-052-RBDtri (Panels M-P). Panels A-G show lung lesions in control SARS-CoV-2 infected rhesus macaques with multifocal interstitial pneumonia, type 2 pneumocyte hyperplasia, alveolar septal thickening, syncytia formation, neutrophils and macrophages infiltrations and severe fibrosis (RVo17). Panels I, K, and L show lesser thickening of alveolar septa, mild perivascular cuffing and decreased type 2 pneumocytes hyperplasia; Panel J shows extensive interstitial pneumonia with serofibrinous exudate. Panels H, O, and P show no significant microscopic lesions. Panels M and N show mild peribronchiolar hyperplasia and perivascular cuffing. This experiment was conducted on lung sections derived from control (n=7), alum (n=5) and alum-3M-052 (n=4) vaccinated animals following SARS-CoV-2 challenge, and multiple sections were scanned for pathological features and then the representative image with 100x magnification is shown. See also supplementary Table 1.

Supplementary Fig. 4

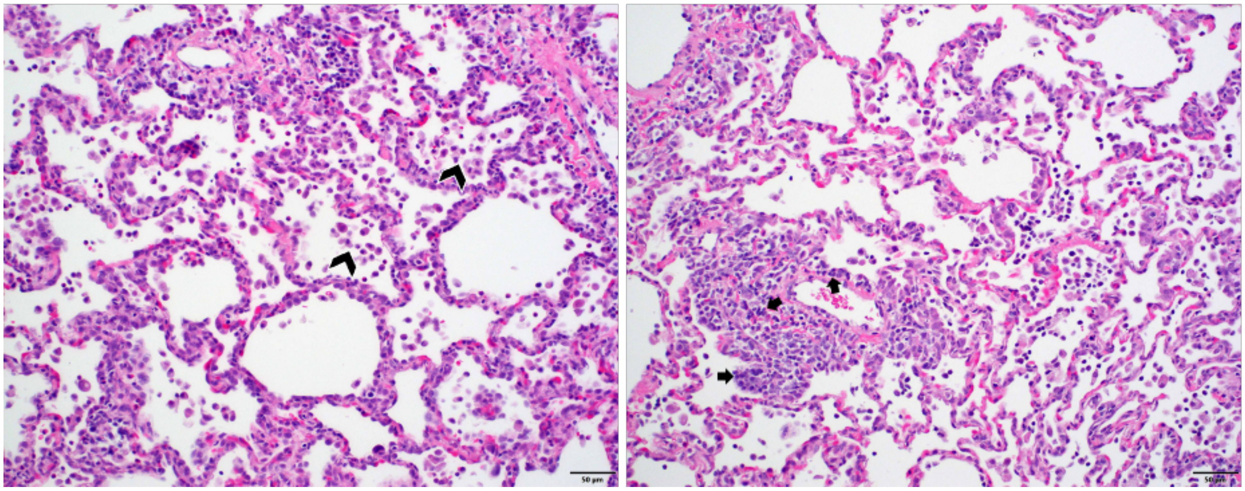

Supplementary Fig. 4: Histopathological examination of neutrophils and macrophages recruitment in lung tissue sections of rhesus macaque. Lung Pathology examination of rhesus macaques infected with SARS-CoV-2. Arrowheads on the left image show clusters of intra-alveolar macrophages for macaque Rup17 and arrows on the right image show examples of neutrophilic infiltrates for macaque RUp17. This experiment was conducted on lung sections derived from control (n=7), alum (n=5) and alum-3M-052 (n=4) vaccinated animals following SARS-CoV-2 challenge, and multiple sections were scanned for pathological features and then the representative image with 200x magnification and 20x objective is shown.

Supplementary Fig. 5

A

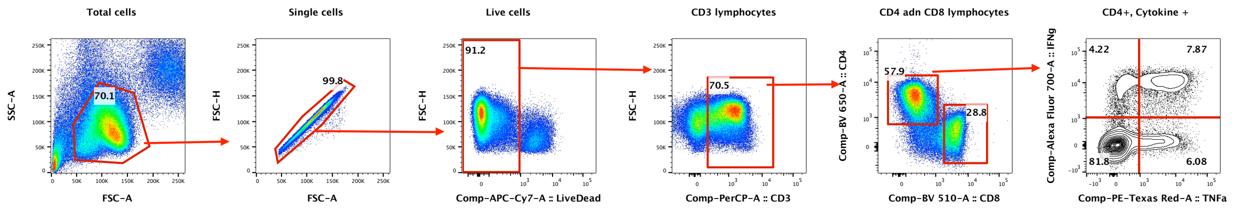

B

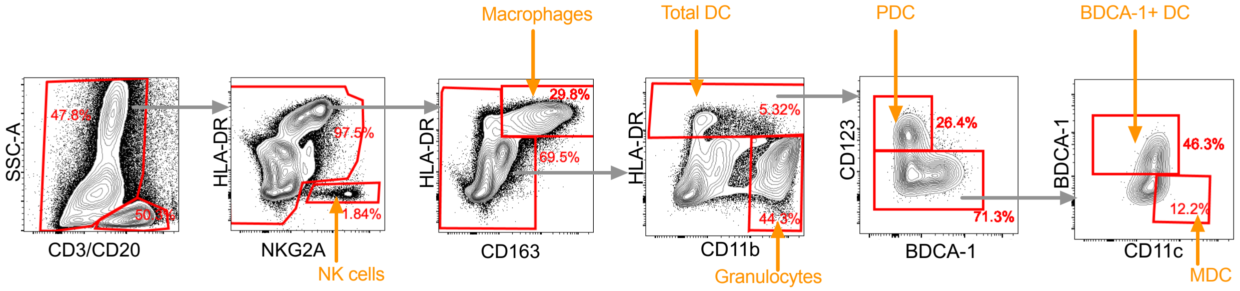

Supplementary Fig. 5: Gating strategy. A) Gating strategy for intracellular staining (ICS) assay in flow analysis. Total lymphocytes (FSC-A and SSC-A) and singlets (FSC-A and FSC-H) were gated using scatter. Live cells were selected using live/dead marker and followed CD3 expression was used to gate T cells. The T cells were then separated into CD4 versus CD8 co-receptor subsets. For CD4 subset, cytokines. Phorbol myristate acetate (PMA) and Ionomycin positive controls. B) Live cells were selected using live/dead marker and $CD3^{+}$ and $CD20^{+}$ cells were excluded. Then, different innate cells were defined using the following combination of markers: NK cells ($HLADR^{-}$ $NKG2A^{+}$), Macrophages ($HLADR^{+}$ $CD163^{+}$); PDCs ($HLADR^{+}CD163^{-}CD123^{+}$ $BDCA1^{-}$); $BDCA1^{+}$ DC ($HLADR^{+}$ $CD163^{-}$ $CD123^{-}$ $BDCA1^{+}$); and MDCs ($HLADR^{+}CD163^{-}CD123^{-}$ $CD11C^{+}$). NK – Natural Killer cells; DC – Dendritic cells; PDCs (Plasmacytoid DCs); and MDCs (Myeloid DCs).

Supplementary Table 1: Lung pathology scores post infection.

	Animal ID	Type 2 pneumocyte hyperplasia	Alveolar septal thickening	Fibrosis	Perivascular cuffing	Peribronchiolar hyperplasia	Syncytia formation	Total score
MVA-Wt (Control)	RTb18	3	2	0	2	2	1	**10**
	RTr17	0	1	0	2	2	1	**6**
	RWb18	2	2	0	2	2	1	**9**
	RVo17	1	1	3	2	1	0	**8**
	RUp17	2	2	0	2	1	0	**7**
Control	RGa17	0	1	0	1	0	0	**2**
	Lc16	1	2	0	2	0	1	**6**
Alum (Vaccine)	33586	0	0	0	0	0	0	**0**
	33593	2	2	0	1	0	1	**6**
	RRk17	0.8	1.4	0	1.4	0	0	**3.6**
	R33395	1	2	0	2	0	1	**6**
	33595	1	2	0	2	0	1	**6**
Alum-3M052 (Vaccine)	34002	0	0	0	1	1	0	**2**
	34514	0.5	0.5	1	0.5	0	0	**2.5**
	34526	0	0	0	0	0	0	**0**
	34532	0	0	0	0	0	0	**0**

Supplementary Table 2: Primers used in this study.

Title	Sequences
SGMRNA-E-Fw	5'-CGATCTCTTGTAGATCTGTTCTC-3'
SGMRNA-E-Rv	5'-ATATTGCAGCAGTACGCACACA-3'
SGMRNA-E-Pr	5'-FAM-ACACTAGCCATCCTTACTGCGCTTCG-3'
RM-RPP30-Fw	5'-AGACTTGGACGTGCGAGCG-3'
RM-RPP30-Rv	5'- GAGCCGCTGTCTCCACAAGT-3'
and RPP30-Pr	5'-FAM-TTCTGACCTGAAGGCTCTGCGCG-BHQ1-3'
