# Supplementary material for: SARS-CoV-2 RBD trimer protein adjuvanted with Alum-3M-052 protects from SARS-CoV-2 infection and immune pathology in the lung
Source: Nat Commun. 2021 Jun 11;12:3587. doi: 10.1038/s41467-021-23942-y (PMC8196016; doi:10.1038/s41467-021-23942-y)
Supplement: Supplementary file 3 — Reporting Summary [file 41467_2021_23942_MOESM3_ESM.pdf]

nature research

Corresponding author(s): Rama Rao Amara

Last updated by author(s): May 16, 2021

Reporting Summary

Nature Research wishes to improve the reproducibility of the work that we publish. This form provides structure for consistency and transparency in reporting. For further information on Nature Research policies, see our Editorial Policies and the Editorial Policy Checklist.

Statistics

For all statistical analyses, confirm that the following items are present in the figure legend, table legend, main text, or Methods section.

n/a	Confirmed	
☐	☒	The exact sample size (*n*) for each experimental group/condition, given as a discrete number and unit of measurement
☐	☒	A statement on whether measurements were taken from distinct samples or whether the same sample was measured repeatedly
☐	☒	The statistical test(s) used AND whether they are one- or two-sided<br>*Only common tests should be described solely by name; describe more complex techniques in the Methods section.*
☐	☒	A description of all covariates tested
☐	☒	A description of any assumptions or corrections, such as tests of normality and adjustment for multiple comparisons
☐	☒	A full description of the statistical parameters including central tendency (e.g. means) or other basic estimates (e.g. regression coefficient) AND variation (e.g. standard deviation) or associated estimates of uncertainty (e.g. confidence intervals)
☐	☒	For null hypothesis testing, the test statistic (e.g. F, t, r) with confidence intervals, effect sizes, degrees of freedom and P value noted<br>*Give P values as exact values whenever suitable.*
☒	☐	For Bayesian analysis, information on the choice of priors and Markov chain Monte Carlo settings
☒	☐	For hierarchical and complex designs, identification of the appropriate level for tests and full reporting of outcomes
☐	☒	Estimates of effect sizes (e.g. Cohen's d, Pearson's r), indicating how they were calculated

Our web collection on statistics for biologists contains articles on many of the points above.

Software and code

Policy information about availability of computer code

Data collection	BD FACSDiva Software v8.0.1, SOFTmaxPro, Image Lab 5.2, CTL ImmunoSpot S6 Universal Analyzer.
Data analysis	GraphPad Prism version 8.4.3 (GraphPad Software), Viridot, Forecyt (v 8.1)

For manuscripts utilizing custom algorithms or software that are central to the research but not yet described in published literature, software must be made available to editors and reviewers. We strongly encourage code deposition in a community repository (e.g. GitHub). See the Nature Research guidelines for submitting code & software for further information.

Data

Policy information about availability of data

All manuscripts must include a data availability statement. This statement should provide the following information, where applicable:

- Accession codes, unique identifiers, or web links for publicly available datasets
- A list of figures that have associated raw data
- A description of any restrictions on data availability

All data supporting the experimental findings of this study are available within the manuscript and are available from the corresponding author upon request.

Field-specific reporting

Life sciences study design

All studies must disclose on these points even when the disclosure is negative.

Sample size	No statistical methods were used to predetermine samples size. For the mouse studies, we have used 5 mice per group and repeated the experiments at least 2 times except for the challenge experiment. This is standard for mouse experiments. For the macaque studies, a total of 16 Indian origin rhesus macaques (Macaca mulatta; male) from 4–6 years of age were included in this study. These were distributed into 3 groups with 4-7 animals/group. We chose this number since recently published NHP studies including our own allowed to see significant difference between vaccinated and control animals for immunogenicity and protection. In addition, the NHP studies are expensive and there is a scarcity for the animal availability so we can't use group sizes that are larger than this.
Data exclusions	No data were excluded.
Replication	For the characterization of constructs, binding data, neutralization data, all experiments are reproducible. The animal experiments and SARS-CoV-2 challenge experiments were not repeated. The in vitro experiments were repeated twice, we have noticed similar observations and antibody related experiments, we have 5 animals (n=5 mice per group) and each sample was analyzed in duplicates and in each experiments representative data are shown. We have conducted SARS-CoV-2 challenge experiments in both mouse and monkey models (were not repeated) and noticed similar observations.
Randomization	We randomly divided monkeys (Macaca mulatta; male) and allocated into groups. However, we did not do this for mouse studies since all mice are genetically identical with same age and source, and we have the opportunity to repeat. Many analyses such as neutralizing antibody, luminex based binding antibody and viral load measurements were done on a blinded fashion.
Blinding	Veterinarians that performed NHP study were blinded to study design. Many analyses such as neutralizing antibody, luminex based binding antibody and viral load measurements were done on a blinded fashion. ELISA based binding antibody assays were not done in a blinded fashion since these were performed by the same individual who performed mouse immunization.

Reporting for specific materials, systems and methods

We require information from authors about some types of materials, experimental systems and methods used in many studies. Here, indicate whether each material, system or method listed is relevant to your study. If you are not sure if a list item applies to your research, read the appropriate section before selecting a response.

Materials & experimental systems

n/a	Involved in the study	
☐	☒	Antibodies
☐	☒	Eukaryotic cell lines
☒	☐	Palaeontology and archaeology
☐	☒	Animals and other organisms
☒	☐	Human research participants
☒	☐	Clinical data
☒	☐	Dual use research of concern

Methods

n/a	Involved in the study	
☒	☐	ChIP-seq
☐	☒	Flow cytometry
☒	☐	MRI-based neuroimaging

Antibodies

Antibodies used	A description of all antibodies used is provided in manuscript. Live/Dead-APC-Cy7, anti-CD3, anti-CD4 and anti-CD8, anti-IFNγ, TNFα, IL-2, IL-21, IL-4 and IL-17. BAL innate cell surface antibody cocktail: live/dead stain-APC-cy7, anti-CD3-605, anti-CD20-605, anti-NKG2A-APC, anti-HLADR-PERCP, anti-cd11b-PE/Dazzle 594, anti-163-eflour-450, anti-CD123-PEcy7, anti-CD11c-BV655 and anti-BDCA1-BV711. BAL innate cell intracellular antibody: anti-Ki67-BV786. T-cell phenotype surface antibody cocktail: live/dead stain-APC-cy7, anti-CD3-PerCP, anti-CD4-BV655, anti-CD8-BV711, anti-PD1-BV421, anti-CXCR5-PE and anti-CXCR3-BV605. T-cell phenotype intracellular antibody: anti-Ki67-BV786. B-Cell phenotype surface antibody cocktail: live/dead stain-APC-cy7, anti-CD3-AF700 and anti-CD20-BV605, B-cell phenotype intracellular antibody: anti-BCL6-PE-CF594 and anti-Ki67-PEcy7. Manufacture recommended volumes per test were used throughout the experiments.
Validation	All antibodies and reagents used in our study were validated by vendors, sources and in our experimental positive controls. All antibodies used are commercially validated on the manufacturer's website as flow cytometry reagents for mouse experiments and either raised against macaque antigens or validated to cross-react with macaque antigens according to the manufacturer's website.

Eukaryotic cell lines

Policy information about cell lines

Cell line source(s)	HEK (Human Embryonic Kidney)-293T cells, and Vero (E6) cells were obtained from ATCC.

Authentication	Acquired from ATCC with Certificate of authentication
Mycoplasma contamination	Negetive
Commonly misidentified lines (See ICLAC register)	None

Animals and other organisms

Policy information about studies involving animals; ARRIVE guidelines recommended for reporting animal research

Laboratory animals	Details for all the animals mentioned in methods. Specific-pathogen-free (SPF) 6–8-week-old female BALB/c mice (00065 strain) were obtained from Jackson Laboratories (Wilmington, MA, USA) and housed in the animal facility at the Yerkes National Primate Research Center of Emory University, Atlanta, GA. Male Indian rhesus macaques (Macaca mulatta), 3-4.5 years old, were housed in pairs in standard non-human primate cages and provided with both standard primate feed (Purina monkey chow) fresh fruit, and enrichment daily, as well free access to water. Immunizations, blood draws, and other sample collections were performed under anesthesia with ketamine (5-10 mg/kg) or telazol (3-5 mg/kg) performed by trained research and veterinary staff. Rodents were maintained on a 12hr light/dark cycle at a temp/humidity: 68-79F/30 – 70% and our animal rooms are monitored electronically through an automated system.
Wild animals	No wild animals were used in the study.
Field-collected samples	No field collected samples were used in the study.
Ethics oversight	The macaque animal study was conducted at Yerkes National Primate Research Center, Emory University, and was approved by the Emory IACUC. All mouse challenge experiments were carried out at the University of North Carolina (UNC) at Chapel Hill.

Note that full information on the approval of the study protocol must also be provided in the manuscript.

Flow Cytometry

Plots

Confirm that:

- [x] The axis labels state the marker and fluorochrome used (e.g. CD4-FITC).
- [x] The axis scales are clearly visible. Include numbers along axes only for bottom left plot of group (a 'group' is an analysis of identical markers).
- [x] All plots are contour plots with outliers or pseudocolor plots.
- [x] A numerical value for number of cells or percentage (with statistics) is provided.

Methodology

Sample preparation	All details related to sample preparation are included or cited in text and methods section. Experiments involving, SD_S-PAGE gels and western blotting, the cells were harvested and lysed in ice-cold RIPA buffer and supernatants were collected. Lysates were kept on ice for 10 min, centrifuged, and resolved by SDS PAGE using precast 4–15% SDS polyacrylamide gels (BioRad).<br>The blood samples were collected at two weeks following each immunization by facial vein puncture in BD Microtainer® Tube for analyzing SARS-CoV-2 RBD-specific serum antibody responses.<br>To prepare serum dilution for ELISA, mouse and NHP sera were incubated at 56°C for 30 min and manually diluted in duplicate in 4% whey powder in DPBS with 0.05% Tween 20 buffer.<br>To prepare serum for Live-virus SARS-CoV-2 neutralization assays, sera were incubated at 56°C for 30 min and manually diluted in duplicate in serum-free Dulbecco's Modified Eagle Medium (DMEM).<br>For macaques, PBMC from blood collected in sodium citrate CPT tubes were isolated using standard procedures. Post SARS-CoV-2 challenge, samples were processed and stained in BSL-3 facility.<br>For BAL fluid processing, and single-cells isolation, up to 50 ml physiological saline was delivered through the trachea into the lungs of anesthetized animals using a camera enabled fiberoptic bronchoscope. The flushed saline was re-aspirated 5 times before pulling out the bronchoscope. This collection was filtered through 70µm cell strainer and centrifuged at 1126 rpm for 5 minutes. Pelleted cells were suspended in 1ml R10 medium (RPMI(1X), 10% FBS) and stained as described in sections below.
Instrument	BD FACSDiva Software v8.0.1, SOFTmaxPro, Image Lab 5.2, CTL ImmunoSpot S6 Universal Analyzer.
Software	GraphPad Prism version 8.4.3 (GraphPad Software), Viridot
Cell population abundance	We represented the frequency of the population and cell number wherever required.
Gating strategy	The gating strategy is represented as it when required in manuscript.<br>Identification of Cytokine positive cells in intra cellular satining (ICS), lymphocytes were identified by FSC-A vs SSC-A gating, followed by doublet exclusion (FSC-A vs FSC-H). Followed by frequencies of various CD3+ cells were identified from % of live cell population. Followed by, used that included CD3, CD4, CD8 and the cytokine (IFNγ, TNFα, IL-2, IL-21, IL-4 and IL-17).<br><br>Identification of Lymphocytes were identified by FSC-A vs SSC-A gating, followed by doublet exclusion (FSC-A vs FSC-H).

Followed by frequencies of various innate cells, Monocytes (HLA-DR+) - classical (CD14+), intermediate (CD14+ and CD16+) and non-classical (CD16+); pDCs (HLADR+ CD14- CD16- CD123+ BDCA1-); BDCA1+ DC (HLADR+ CD14- CD16- CD123- BDCA1+); mDCs (HLADR+ CD14- CD16- CD123- CD11c+); M-MDSC cells (HLADR- CD14+ CD11b+); PMN-MDSC cells (HLADR- CD14- CD11b+) and NK cells (HLADR- NKG2A+); and CD86+ activation.

☒ Tick this box to confirm that a figure exemplifying the gating strategy is provided in the Supplementary Information.
